# ExAblate neuro transcranial treatment considerations

**DOI:** 10.1186/2050-5736-3-S1-O33

**Published:** 2015-06-30

**Authors:** Eyal Zadicario

**Affiliations:** 1 InSightec, Tirat Carmel, Israel

## Background/introduction

ExAblate Neuro is the only technology that enables clinical treatment of transcranial MR guided focused ultrasound. It is CE approved for the treatment of certain neurological disorders and is under evaluation for additional regulatory approval.

We will review the existing applications and the limitations of the current technology. Two of the key limitations are the treatment envelope and the effects of different skull on the effectiveness of the transcranial ultrasound delivery.

## Methods

Expanding the treatment envelope requires to overcome technological barriers. The current clinical experience is limited to anatomical targets that are limited to deep and centralized locations in the brain. This is a significant limitation since there are compelling clinical targets that are located beyond the existing treatment envelope. We will suggest treatment concepts which are being developed to overcome these limitations and enable to extend the treatment envelope.

## Results and conclusions

The accumulated clinical experience with transcranial focusing shows as significant variability of the acoustic parameters which are applied during the treatments. It is assumed that the skull is the most significant contributor to this variability due to its non-homogenous and quite variable characteristics. The variability has been studied and found to be correlated with skull density parameters. We will describe how patient selection criteria may be applied to optimize the treatment for different patient selection criteria.

